# Soft-Error-Resilient Static Random Access Memory with Enhanced Write Ability for Radiation Environments

**DOI:** 10.3390/mi16111212

**Published:** 2025-10-24

**Authors:** Se-Yeon Park, Eun Gyo Jeong, Sung-Hun Jo

**Affiliations:** 1 Department of Semiconductor Engineering, Tech University of Korea, Siheung 15073, Republic of Korea; goofysis@tukorea.ac.kr; 2 Department of Electronics Engineering, Incheon National University (INU), 119 Academy-ro, Yeonsu-gu, Incheon 22012, Republic of Korea; 3 Division of System Semiconductor, Dongguk University, Seoul 04620, Republic of Korea

**Keywords:** critical charge, single-event-multi-node-upset (SEMNU), single event upset (SEU), write access time, write stability

## Abstract

As semiconductor technologies advance, SRAM cells deployed in space systems face heightened sensitivity to radiation-induced soft errors. In conventional 6T SRAM, when high-energy particles strike sensitive nodes, single-event upsets (SEUs) may occur, flipping stored bits. Furthermore, with aggressive scaling, charge sharing among adjacent devices can trigger single-event multi-node upsets (SEMNU). To address these reliability concerns, this study presents a radiation-hardened SRAM design, SHWA18T, tailored for space applications. The proposed architecture is evaluated against IASE16T, PRO14T, PRO16T, QCCS, SIRI, and SEA14T. Simulation analysis demonstrates that SHWA18T achieves improved performance, particularly in terms of critical charge and write capability. The design was implemented in 90 nm CMOS technology at a 1 V supply. With enhanced robustness, the cell withstands both SEUs and SEMNUs, thereby guaranteeing stable data retention in space environments.

## 1. Introduction

Electronics are increasingly deployed in harsh radiation environments, particularly in space. Satellite communication, in particular, plays an essential role in global infrastructure. With advances in technology, lightweight satellites are being developed to minimize costs associated with production, launch, and maintenance [1]. Such satellites demand memory architectures with both compactness and high density. SRAM has been widely adopted in space because of its density and speed. However, aggressive technology scaling has led to reduced supply voltage, smaller critical charge, and denser transistors, which have made SRAM cells more prone to failures.

These failures largely stem from radiation-induced soft errors. The most common fault is the single-event upset (SEU), in which stored information is temporarily flipped. SEU occurs more frequently in SRAM cells with a high density of integration, low critical charge, and low power supply voltage [2]. Such upsets occur when a high-energy particle ionizes atoms within a semiconductor, producing electron–hole pairs. Due to the reverse-bias field, these carriers separate, and the resulting charge accumulation generates a transient voltage pulse, or single-event transient (SET). If this SET has sufficient amplitude and duration, it can disturb stored states in memory cells, latches, or flip-flops, leading to SEUs.

SRAM is more vulnerable to SEUs than other memory types because of its large sensitive area and low capacitance. Additionally, reduced device spacing with continued scaling increases the probability of single-event multi-node upsets (SEMNU), where one ion strike perturbs multiple nodes. For this reason, circuit-level reinforcement of SRAM cells is essential. Radiation-hardening strategies allow cells to recover from both SEUs and SEMNUs.

The maximum operating frequency of the Radiation-Hardened SRAM depends on the operating speed of the memory cell’s critical path delay, sense amplifier, word line driver, bit-line precharge, and the applied radiation-hardened structure complexity. For example, as the number of transistors in memory cells increases, the number of internal nodes increases, so driving capacity decreases and parasitic capacitance increases, which tends to increase read and write latency. In general, in the case of Radiation-Hardened SRAM, the maximum operating frequency may be lower than that of regular commercial SRAM because the number and size of transistors are increased to ensure stability and reliability and wide spacing between nodes. Furthermore, Cosmic radiation can induce variations in the threshold voltage and carrier mobility of transistors, which may lead to reduced circuit speed and, consequently, impact the operating frequency.

Approaches such as triple modular redundancy (TMR), error-correcting codes (ECC), and hardened topologies have been proposed. TMR provides robustness but requires a large area and power, while ECC involves extra encoder/decoder logic, creating overhead [3]. Circuit-level designs, therefore, offer a more efficient solution. Standard 6T SRAM cannot recover from SEUs, as voltage perturbations propagate between cross-coupled nodes. Hardened designs including IASE16T [4], PRO14T [5], PRO16T [5], and QCCS [6] improve SEU tolerance, while SEA14T [7] and SIRI [8] were developed for SEMNU mitigation. This paper proposes SHWA18T, an SRAM design capable of recovering from SEUs at any sensitive node and from SEMNU disturbances across internal node pairs. SHWA18T has two additional access transistors for improving the write operation [7]. Increased access to transistors accelerates operation speeds, such as read or write operations.

## 2. State-of-the-Art of Soft-Error-Resilient SRAM

A standard 6T SRAM cell employs a pair of cross-coupled inverters that form a positive feedback loop. When an energetic radiation particle strikes a vulnerable node, the stored logic value can be flipped once the deposited charge surpasses the critical threshold. Because of the feedback connection, the disturbance may propagate to the complementary storage node, leading to instability in the entire cell. Hence, conventional 6T designs are highly susceptible to radiation-induced faults. To overcome these weaknesses, various radiation-hardened cell structures have been introduced, including IASE16T, PRO14T, PRO16T, QCCS, SEA14T and SIRI.

**Figure 1:**
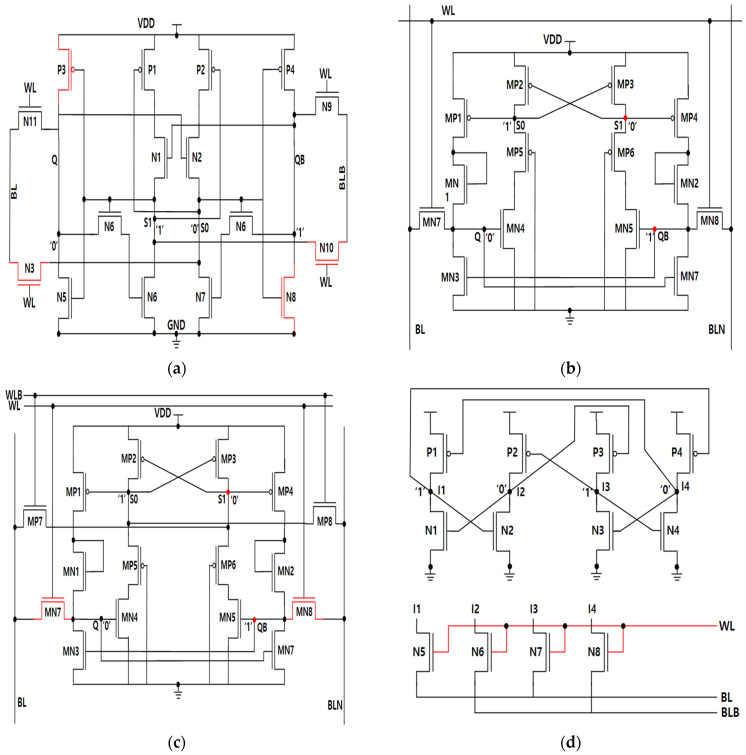

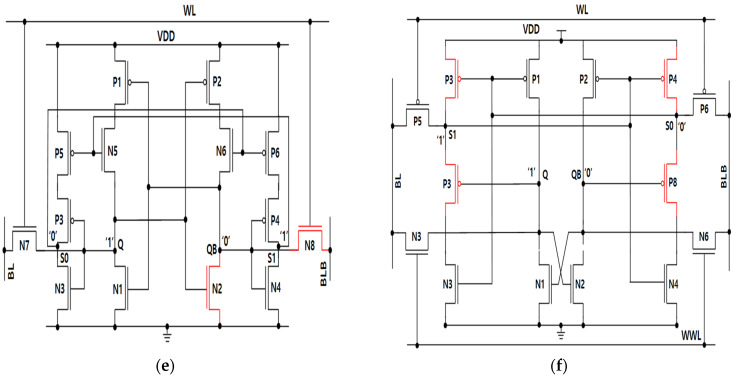
State-of-the-Art of reliable SRAM memory cells (VDD = 1V) (**a**) IASE16T, (**b**) PRO14T, (**c**) PRO16T, (**d**) QCCS, (**e**) SEA14T; (**f**) SIRI.

In the IASE16T cell, a fault-tolerant recovery scheme is incorporated at all vulnerable nodes to achieve full resilience and functional restoration following an SEU event, as depicted in Figure 1a. Its major strength lies in excellent hold stability. However, the design cannot autonomously recover from SEMNU events, and its critical charge is lower compared with other hardened structures. In the case of the PRO14T cell (Figure 1b), two sensitive nodes are utilized. The main advantage of this configuration is the reduced probability of SEU generation, owing to the dual-node arrangement. Nonetheless, similar to IASE16T, the cell is incapable of SEMNU recovery and also suffers from a relatively small critical charge. The PRO16T structure (Figure 1c) is an extension of the PRO14T cell, incorporating two additional transistors while maintaining two sensitive nodes. This modification enhances the performance and provides a larger write margin, though at the cost of slightly increased power consumption. The major drawbacks, however, remain unchanged, such as low critical charge and inability to recover SEMNU. As shown in Figure 1d, the QCCS architecture employs four storage nodes interconnected in a large feedback loop. Its most favorable characteristics include superior hold stability and an improved critical charge compared to conventional cells. The disadvantages, however, are its limited write ability and lack of robustness against DNU disturbances. The SIRI cell (Figure 1e) contains three sensitive nodes. Owing to its isolated storage nodes from the bit-lines, this structure exhibits the highest read stability among the compared designs and consumes the lowest hold power. Furthermore, SEMNU can be recovered in the Q–QB node pair. Nevertheless, the penalty is an increased read delay and reduced write capability. Finally, the SEA14T cell, illustrated in Figure 1f, incorporates three sensitive nodes. Its standout benefits include high write ability, low hold power consumption, and the largest critical charge among all evaluated cells. In addition, it enables DNU recovery in the S1–S0 node pair. The limitation, however, is that only one specific node pair can be restored after a DNU event.

## 3. Proposed SHWA18T SRAM Cell

**Figure 2:**
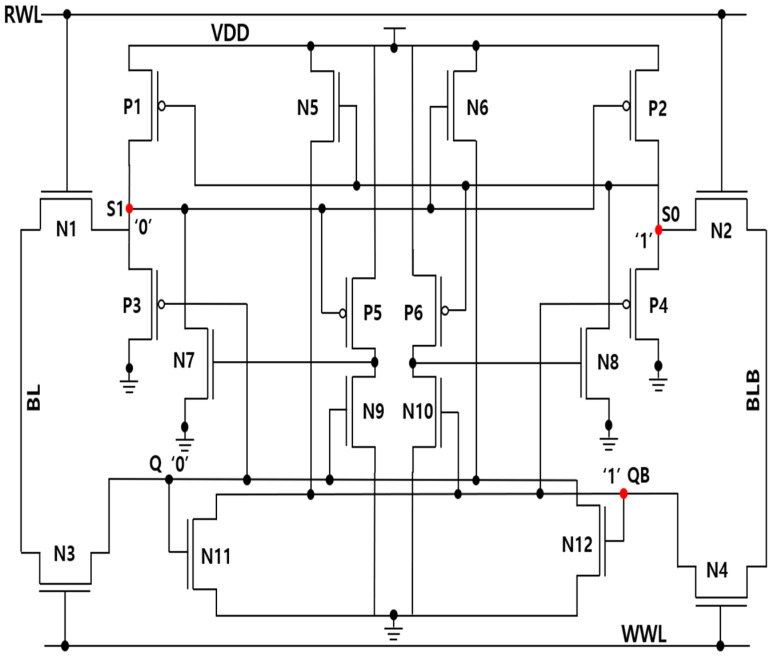
Schematic of the proposed SHWA18T cell (VDD = 1 V).

**Figure 3:**
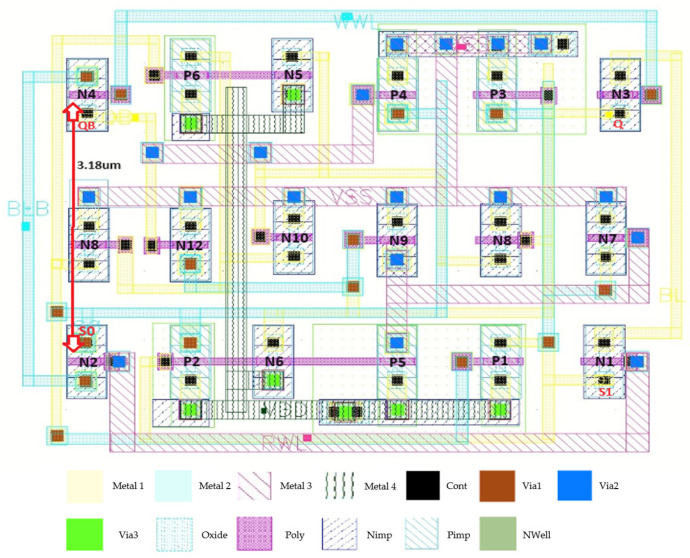
Layout of the proposed SHWA18T cell.

The schematic of the SHWA18T cell is illustrated in Figure 2, and its layout is provided in Figure 3. As depicted in Figure 2, the memory cell incorporates four NMOS access transistors (N1–N4) to enhance read performance. Specifically, the read wordline (RWL) drives transistors N1 and N2, thereby linking the internal nodes (S0 and S1) to the corresponding bit-lines (BL and BLB). Similarly, the write wordline (WWL) activates transistors N3 and N4, which establish the connection between the storage nodes (Q and QB) and the bit-lines, improving the writing capability. The SHWA18T design consists of two primary storage nodes, Q and QB, along with two auxiliary internal nodes, S0 and S1. Considering the case where the cell stores logic ‘0’ nodes Q and S1 maintain a value of ‘0’ while nodes QB and S0 retain a value of ‘1’.

### 3.1. Operation of SHWA18T Cell

#### 3.1.1. Hold Operation

During the hold state, both the read and write wordlines (RWL and WWL) are kept inactive, which ensures that all access transistors are switched off. At the same time, the bit-lines are precharged to VDD in order to minimize the delay when the cell transitions out of the hold mode [9]. Under these conditions, transistors P2, P4, P5, and N5 remain conducting, while the remaining devices are turned off, thereby maintaining the data that was originally stored.

#### 3.1.2. Read Operation

In the read operation, the bit-lines are precharged to VDD, and the read wordline (RWL) is driven high to VDD. Once transistors N1 and N2 are enabled, the BLB line starts to discharge through transistors N6 and N8. This discharging continues as long as N6 and N8 remain conducting. Since N5 and N7 are turned off, the BL line holds its precharged value at VDD. When the potential difference between BL and BLB becomes about 50 mV, the sense amplifier turns on and reads out the corresponding information.

#### 3.1.3. Write Operation

For writing complementary values into the cell, the bit-lines BL and BLB are biased to GND and VDD, respectively, while both the read and write wordlines (RWL and WWL) are enabled. As a result, nodes Q and S1 are pulled to ground, and QB and S0 are driven to VDD through their corresponding bit-lines. For instance, when changing the stored state from logic ‘0’ to logic ‘1’, BL is raised to VDD and BLB is tied to the supply voltage. Under these conditions, the storage node Q is charged through transistor N3, whereas node QB is discharged via transistor N10. With N10 conducting, QB is consequently forced to logic ‘0’.

### 3.2. Soft Error Recovery Analysis

When a node is placed adjacent to a reverse-biased drain diffusion region, it is regarded as sensitive because incoming energetic particles are more likely to induce charge collection. This collected charge can lead to a short-term change in the stored value, and the exact effect depends on whether the node is connected to a PMOS or an NMOS device. If the node is linked to a PMOS (NMOS) device, the transient upset generally manifests as a ‘0→1’ (‘1→0’) flip, provided the initial logic level allows such a transition. In the case of the Q node storing logic ‘0,’ no state change occurs (‘0→0’) because it is only surrounded by NMOS transistors, as shown in Figure 2. By contrast, the QB node holding logic ‘1’ undergoes a ‘1→0’ transition for the same reason, making it the sole sensitive node among the storage nodes. Assuming the memory cell stores logic ‘0,’ the values held at Q, QB, S0, and S1 are 0, 1, 1, and 0, respectively.

#### 3.2.1. SEU at QB

**Figure 4:**
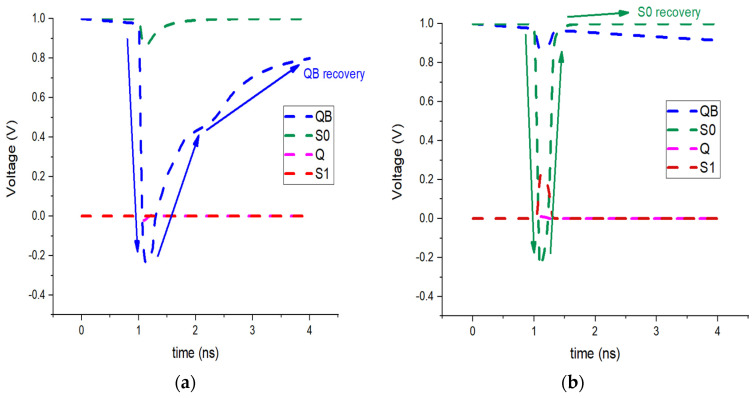

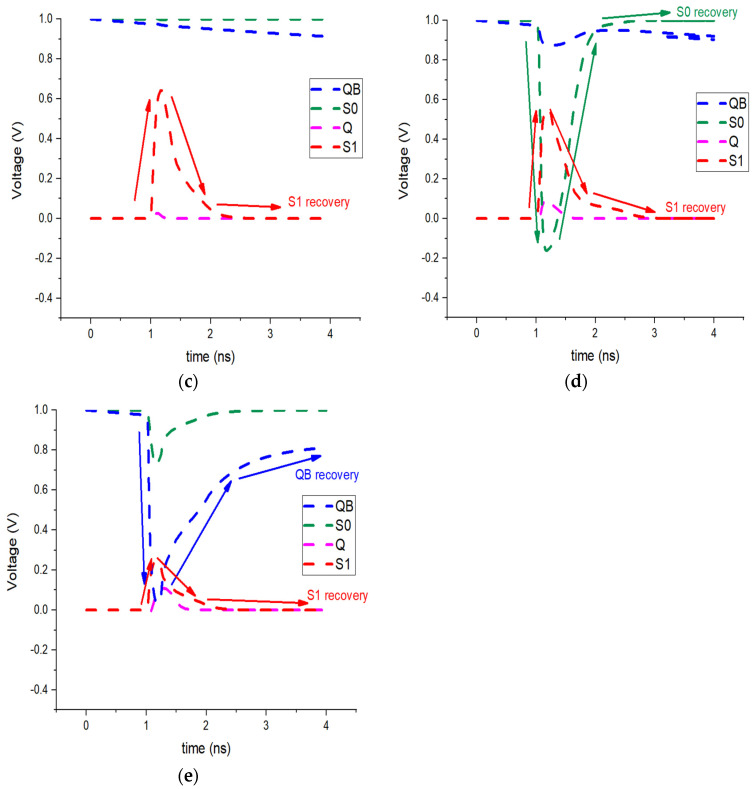
SEU recovery of SHWA18T when SEU affects (**a**) node QB, (**b**) node S0, (**c**) node S1, (**d**) node pair (S0-S1) and (**e**) the node pair (QB-S1) simultaneously.

If a high-energy particle interacts with the storage node QB, the logic level can momentarily shift from ‘1’ to ‘0.’ As a consequence, P3, N10, and N12 are turned off, leaving nodes Q, S0, and S1 floating in a high-impedance state. According to findings in [10], this condition does not alter the logic contents of those nodes, even under particle strikes. Hence, Q, S0, and S1 preserve their original logic states, with S0 initially storing a ‘1’ and S1 a ‘0.’ Transistor N5 then restores QB to its correct value of ‘1.’ This recovery is depicted in Figure 4a, where an SEU event is shown at QB.

#### 3.2.2. SEU at S0

A particle impact on the S0 internal node can cause a transient flip of its stored data from logic ‘1’ to logic ‘0.’ As a result, transistor P6 becomes active for a short time. The other storage nodes—Q, QB, and S1—remain unchanged, keeping values of 0, 1, and 0, respectively. Because S0 is supported by one pull-up and two pull-down paths, the conduction of transistor P2 restores the node to logic ‘1.’ This recovery process under SEU conditions is shown in Figure 4b.

#### 3.2.3. SEU at S1

If node S1 is hit by an energetic particle, the stored bit may change from ‘0’ to ‘1.’ In this situation, the PMOS transistor P5 is deactivated, and the NMOS transistor N6 becomes conductive. The other nodes remain unaffected: Q stores ‘0,’ QB holds ‘1,’ and S0 keeps ‘1.’ Since P3 and N7 continue operating in the ON state, they provide the recovery path that drives S1 back to logic ‘0.’ The sequence of this SEU event at S1 is shown in Figure 4c.

#### 3.2.4. DNU at Node Pairs QB-S1

An impact from a high-energy particle on the node pair QB and S1 can induce a short-lived disturbance, causing QB to switch from ‘1’ to ‘0.’ In this process, transistors N6, P4, P5, and N10 temporarily change their operating states. Despite this upset, the remaining storage nodes remain stable, with Q retaining ‘0’ and S0 maintaining ‘1.’ Under these conditions, P3 and N7 act to restore S1 to logic ‘0,’ while N5 drives QB back to logic ‘1.’ Consequently, the original values of QB and S1 are reestablished, as illustrated in Figure 4d.

#### 3.2.5. DNU at Node Pairs S0–S1

If the internal pair of nodes S0 and S1 is struck by a high-energy particle, S0 can temporarily shift from logic ‘0’ to ‘1,’ while S1 transitions from ‘1’ to ‘0.’ In this transient state, transistors P1, P6, and N6 conduct, whereas P2, P5, and N5 are turned off. The main storage nodes Q and QB remain stable, holding ‘0’ and ‘1,’ respectively. Recovery occurs as P3 and N7 force S1 back to logic ‘0,’ while P2 restores S0 to logic ‘1.’ Consequently, the original values of S0 and S1 are reestablished, as illustrated in Figure 4e. Based on this recovery sequence, SHWA18T demonstrates the capability to restore stored data when exposed to SEUs at QB, S1, or S0, as well as SEMNUs affecting QB–S1 and S0–S1. Even though SEMNUs may still flip bits, the layout, shown in Figure 3, mitigates these effects by spacing the drain terminals of NMOS transistors N2 and N4 at least 1.62 µm apart. In addition, the fabrication process employs symmetrical device placement and wiring to maintain layout uniformity.

## 4. Simulation and Analysis

To verify the effectiveness of the SHWA18T structure, simulations were performed using a 90 nm CMOS process. The outcomes were benchmarked against other radiation-hardened cells, including IASE16T, PRO14T, PRO16T, QCCS, SEA14T and SIRI. In these comparisons, transistor ratios for each design were set in accordance with the specifications described in their respective publications. The most prominent gain observed was in write access time, which is critical because SRAM cells typically function in write mode. A shorter access time in this state directly enhances the cell’s write capability.

### 4.1. Comparison of Write Delay and Stability

**Figure 5:**
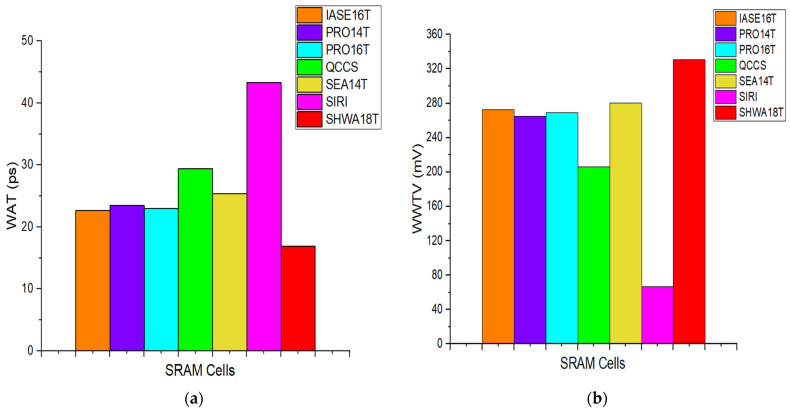
Comparison of the (**a**) WAT and (**b**) WWTV with previous works.

Write access time, which is one of the most important performances of the writing operation, is measured as the interval between the crossing of the storage nodes and the point at which the wordline voltage reaches 50% of its full swing [11]. This timing is mainly determined by how quickly the storage nodes discharge and how strongly the driving transistors enforce the write operation. For a cell storing ‘0,’ the SIRI structure requires a relatively long discharge time because of its extended feedback connections, leading to poor write performance. On the other hand, the PRO14T structure demonstrates a faster WAT, as the pull-up of the internal node initially at logic ‘1’ weakens when QB increases, making discharging more efficient. Nevertheless, in PRO14T, conflicts between pull-up and pull-down devices can slow the node’s discharge process. In comparison, SHWA18T, IASE16T, SEA14T, and PRO16T achieve shorter WATs because their extra access transistors allow internal and storage nodes to change states simultaneously. Among these, SHWA18T and IASE16T stand out with even shorter delays, although IASE16T is somewhat slower than SHWA18T because its QB node strongly stores ‘1’ through a PMOS pull-up (Figure 5a). Another way to quantify write strength is by evaluating the wordline write trip voltage (WWTV) [12,13]. In this method, data is forced on the bit-lines and the wordline voltage is gradually increased. WWTV refers to the voltage offset measured between VDD and the wordline at the instant when the storage nodes cross each other [12,14]. If a cell needs more time to flip, the WL voltage must rise higher, meaning that a larger WAT indicates weaker write ability. Thus, the ranking of WWTV among the compared cells (Figure 5b) is the opposite of that of WAT.

### 4.2. Stability Simulation and Comparison

**Figure 6:**
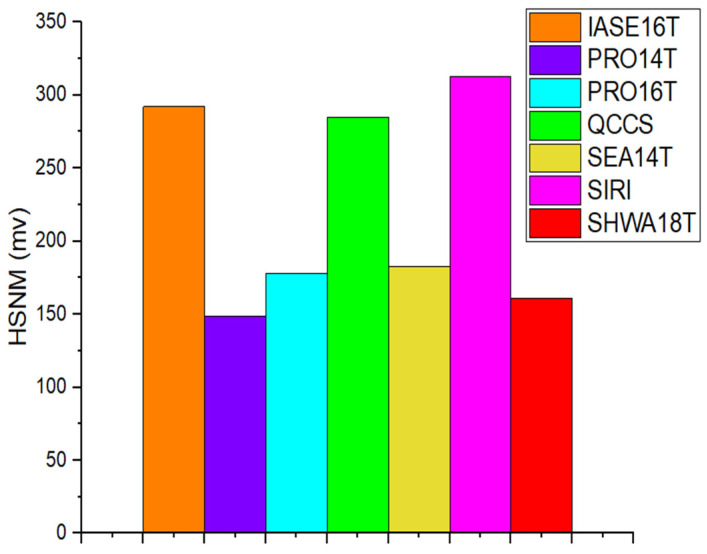
HSNMs among comparison cells.

**Figure 7:**
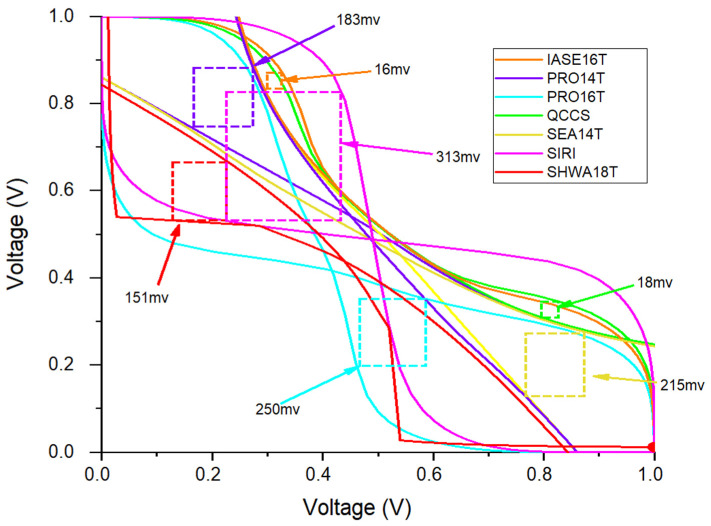
RSNMs among comparison cells.

The static noise margin (SNM) is widely applied as a reliability indicator for SRAM cells [15,16], and is divided into hold SNM (HSNM) and read SNM (RSNM). As illustrated in Figure 6, the HSNM of PRO14T, PRO16T, SEA14T, and SHWA18T are nearly the same, whereas IASE16T, QCCS, and SIRI show stronger HSNM values due to their larger feedback structures. RSNM, on the other hand, measures a cell’s capability to preserve its logic during read access. As shown in Figure 7, following the procedure in [17], PRO16T and SIRI display better RSNM characteristics. In the SIRI architecture, high read stability is obtained because the storage nodes are disconnected from the bit-lines throughout the read phase. PRO16T enhances RSNM by increasing the CR value of the transistor that discharges when BL is tied to ground. In contrast, SHWA18T, despite employing two access devices per bitline, demonstrates lower RSNM (Figure 7). This is because the bit-lines are disconnected from the storage nodes during reading, and the design incorporates two pull-down paths. In practice, only the ‘0’-storing node (S1) shows a voltage rise when accessed, while the states of Q and QB remain unchanged even if S1′s voltage increases significantly.

### 4.3. SEU and SEMNU Immunity Comparisons

**Figure 8:**
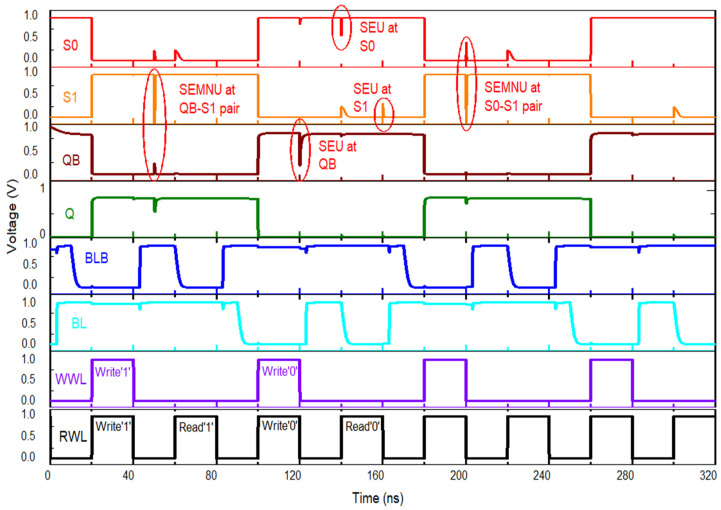
Operations and soft-error recovery of SHWA18T.

SEU immunity can be quantified by evaluating the critical charge ($Q_{C}$), and the corresponding values for different SRAM cells are listed in Table 1. By definition, $Q_{C}$ is the smallest charge that, when collected at a sensitive node, can invert the stored information, and thus it is widely used as a measure of memory robustness. The behavior of SHWA18T under charge injection is presented in Figure 8. According to simulation results, depositing 26 fC of charge at QB, S0, or S1, as well as at the combined pairs {QB–S1} and {S0–S1}, does not cause permanent errors; all nodes are able to restore their original logic levels after the disturbance [18].

(1) $$I\left(t\right)=I_{0}(e^{\frac{-t}{\tau_{\alpha }}}-e^{\frac{-t}{\tau_{\beta }}})$$

(2) $$I_{0}=Q/(\tau_{\alpha }-\tau_{\beta })$$

In this paper, I_0_ refers to the maximum current amplitude, and Q corresponds to the amount of charge deposited in the sensitive node. $\tau_{\alpha }$ is the collection time constant of a junction, $\tau_{\beta }$ is the initial ion track establishing time constant [1]. The current waveform is modeled using a double-exponential function with time constants $\tau_{\alpha }$ = 200 ps and $\tau_{\beta }$ = 50 ps [19]. The critical charge, $Q_{C}$, represents the minimum charge necessary for an SEU to flip the data stored in the most sensitive node. To determine this value, I_0_ is progressively increased until toggling occurs at nodes S0 and S1, and the corresponding total injected charge is measured. Figure 8 presents the simulation of SEU and SEMNU events at the recoverable node pair (S0–S1) of SHWA18T. The design is also able to restore the QB–S1 pair, thereby recovering two of its three sensitive nodes from double-node upsets. By comparison, other hardened cells such as SIRI and SEA14T also employ three sensitive nodes, but they can recover only one pair in the case of a DNU.

### 4.4. Power Dissipation

**Figure 9:**
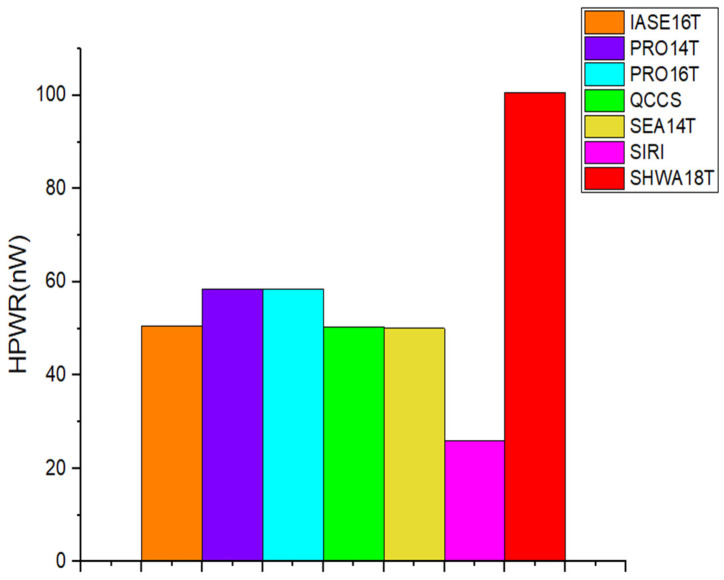
Comparison of the HPWR with previous works.

When an SRAM cell is in its hold state, the static power it dissipates is largely determined by leakage currents within the inverter pairs and along the bit-lines [20]. The detailed measurements of this hold power are presented in Figure 9. The PRO16T and SHWA18T cells show increased HPWR values because their four access transistors introduce additional paths for leakage through the bit-lines. On the other hand, SIRI and IASE16T record lower HPWR, as their design includes triple-transistor stacks that effectively suppress leakage. In addition, SEA14T, having two access transistors, introduces another route for current leakage from the bit-lines.

### 4.5. New Electrical Quality Metric

Several critical performance indicators are commonly used to evaluate SRAM cells, including HSNM, RSNM, $Q_{C}$, WAT, WWTV, and HPWR. Table 2 presents the results of these measurements across different cell structures. The Double-Node Upset Recovery (DNUR) rate reported in the same table indicates the likelihood that a cell can successfully recover from a DNU fault. As previously discussed, SHWA18T exhibits the highest DNUR rate compared with the other candidates. In addition, the area occupied by the cell can be defined by the number of transistors (T). For a holistic evaluation, this work introduces a new figure of merit called the new electrical quality metric (NEQM), which incorporates RSNM, HSNM, $Q_{C}$, WWTV, T, WAT, HPWR, and DNUR [1,21]. A higher NEQM score signifies better global performance of an SRAM cell. NEQM can be defined as follows:

(3) $$NEQM=\frac{RSNM\times HSNM\times Q_{C}\times WWTV\times T}{WAT\times HPWR\times (1-R_{D})}$$

**Figure 10:**
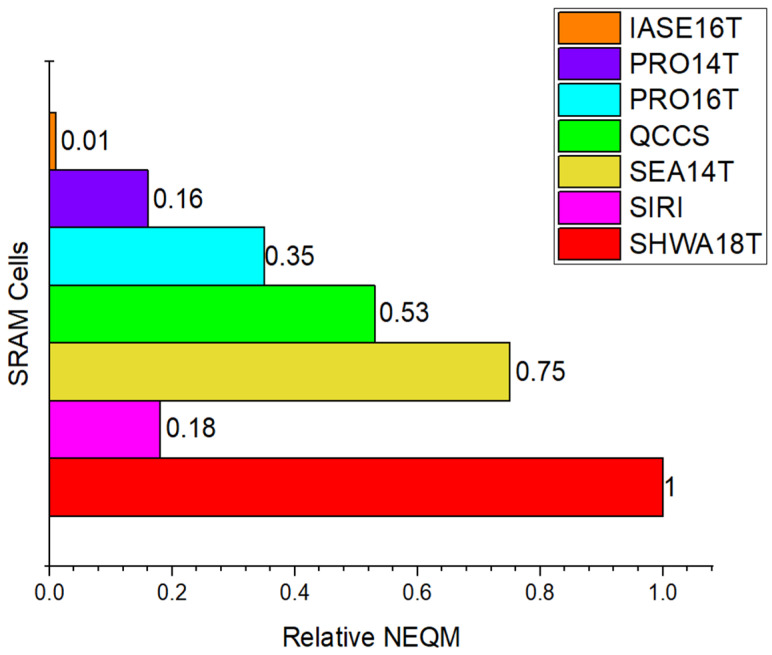
Relative NEQM of different SRAM cells.

To more intuitively compare the NEQM of different SRAM cells, Figure 10 represents the relative NEQM (${NEQM}_{SRAM\;cells}/{NEQM}_{SHWA18T}$). From Figure 10, it can be observed that SHWA18T has the highest NEQM. The NEQM of PRO14T and SIRI are approximately 20% of the SHWA18T, PRO16T is approximately 35% of the SHWA18T, QCCS is approximately 53% of the SHWA18T, SEA14T is approximately 75% of SHWA18T, while the NEQM of IASE16T is under 1% of SHWA18T. The NEQM of other SRAM cells is relatively small in comparison to the SHWA18T. These results indicate that the SHWA18T cell achieves superior reliability and enhanced overall performance compared to conventional designs.

## 5. Conclusions

The SHWA18T cell, a soft-error-tolerant SRAM structure with enhanced write capability, is proposed for use in space systems. All nodes vulnerable to SEUs exhibit strong resistance to single-event upsets and show a high likelihood of recovering from DNUs. In addition, residual fault cases were considered during layout optimization. Compared with other benchmark designs, SHWA18T provides both the greatest write ability and the lowest write access time. It also records a higher critical charge than most of the reference cells. Furthermore, SHWA18T attains the highest NEQM value, which comprehensively reflects its superior overall performance among the evaluated designs.

## Figures and Tables

**Table 1:** Comparisons of critical charge ($Q_{C}$).

Cell	$\mathrm{Effective}\;Q_{C}$ (fC)
IASE16T [4]	10
PRO14T [5]	16
PRO16T [5]	12
QCCS [6]	38
SEA14T [7]	>40
SIRI [8]	10
SHWA18T	26

**Table 2:** Comparison of overall performance metrics.

Design	Ref.	RSNM (mV)	HSNM (mV)	$Q_{C}$ (fC)	WWTV (mV)	Number of Transistors	WAT (ps)	$H_{PWR}$ (nW)	DNUR Rate	Sensitive Node
IASE16T	[4]	16	292	10	273	16	22.7	50.6	0	3
PRO14T	[5]	183	149	12	265	14	23.5	58.5	0	2
PRO16T	[5]	250	178	16	269	16	23	58.5	0	2
QCCS	[6]	190	285	38	206	12	29.39	50.4	0	3
SEA14T	[7]	203	183	>40	280	14	25.4	60.4	33%	3
SIRI	[8]	292	313	10	67	14	43.32	25.89	33%	3
SHWA18T	Proposed	151	161	26	331	18	16.9	100.681	66%	3

## Data Availability

The original contributions presented in this study are included in the article. Further inquiries can be directed to the corresponding author.
